# Headache in the paediatric population: the role of the ophthalmologist

**DOI:** 10.3389/fped.2025.1547750

**Published:** 2025-06-12

**Authors:** Marta Degrassi, Stefania Tonetto, Paola Michieletto, Paolo Dalena, Egidio Barbi, Stefano Pensiero

**Affiliations:** ^1^ Department of Medicine, Surgery, and Health Sciences, University of Trieste, Trieste, Italy; ^2^ Department of Ophthalmology, Institute for Maternal and Child Health—IRCCS “Burlo Garofolo”, Trieste, Italy; ^3^ University of Trieste, Trieste, Italy; ^4^ Institute for Maternal and Child Health—IRCCS “Burlo Garofolo”, Trieste, Italy

**Keywords:** pediatric, headache, ophthalmology, refractive error, strabismus and Pediatric ophthalmology, optic nerve sheath diameter (ONSD), intracranial hypertension, migraine

## Abstract

**Introduction:**

Headache is a very common pathology in pediatric age, but the responsibility of ophthalmological factors in determining headache may be underestimated.

**Aim:**

Identify how headache presents and determine the prevalence of the different causes in children; investigate the role of the ophthalmologist and of the ophthalmic diagnostic investigations in the diagnosis and treatment of pediatric headache.

**Methods:**

101 children, aged 4–18 years, with non-traumatic headache were included in the study. Each child underwent a questionnaire regarding headache characteristics and all the clinical and instrumental examinations necessary to reach the diagnosis.

**Results:**

Migraine was the most common form (28.7%), followed by headache associated with inadequate refractive error. (HARE, 23.7%), tension-type headache (17.8%), strabismus (16.8%), intracranial hypertension (6.9%), digital eye strain (5.9%). Myopia was the most prevalent type of HARE (50%). Strabismus headache was present especially in intermittent exotropia (41.2%) and convergence insufficiency (35.3%).

**Discussion:**

HARE and strabismus, despite the latter is not included in the final version of the International Classification of Headache Disorders, can cause frontal headache in many cases.

**Conclusion:**

Ocular causes are a frequent cause of a relevant percentage of pediatric headaches.

## 1 Introduction

Headache is a frequent condition that strongly impacts daily life of children, is more frequent in females than males, and its prevalence increases with age (1). It is estimated that by age 15, 57%–82% of children will have experienced some form of headache (2). Headache leads to poor health-related quality of life, school attendance and social functioning (1). The causal classification distinguishes primary and secondary forms of headache.

The most frequent forms of primary headache among the pediatric population are migraine and tension-type headache (TTH); migraine has a prevalence of 11%, and tension-type headache (TTH) has a prevalence of 17%, while other primary forms are not well studied in the pediatric population. Thus, more epidemiological data are needed (3).

In pediatrics, ophthalmologists are not frequently involved in the management of headache; however, there are several types of secondary headaches—and possibly even some primary forms—where their expertise could be necessary for better management.

As a matter of fact, even in the primary forms there may be associations with eye problems such as in migraine where, excluding the transient problems during the aura, adults seem to have an augmented risk of glaucoma and its complications (4, 5).

Regarding secondary headaches, the most frequent cause are respiratory tract infections and minor head trauma (6). However, ophthalmologic causes are also reported. The International Classification of Headache Disorders (ICHD) identifies four categories of secondary headache associated with ophthalmological issues: headache attributed to refractive error (HARE), trochlear headache, headache attributed to acute angle-closure glaucoma, and headache attributed to ocular inflammatory disorder (7). Although not listed in the ICHD, some forms of strabismus (8) can cause headaches, mainly convergence defects (9) and accommodative spasms (10). In addition, following the Covid-19 pandemic, the number of pediatric patients complaining of headaches due to the digital eye strain (DES) has increased significantly, demonstrating that the use of electronic devices should also be included among the ophthalmological causes of headaches (11).

Generally, ophthalmologists are involved in managing headaches secondary to intracranial hypertension, a condition suspected when the optic disc is edematous (7).

Although ophthalmologists could play an important role in identifying potential secondary causes of headache and addressing them when present, current literature lacks clear guidelines regarding their involvement in pediatrics. Furthermore, while relatively recent ophthalmologic techniques—such as optic nerve ultrasound and optical coherence tomography (OCT)—have become available for use in pediatric populations over the past decade, their utility in the evaluation of pediatric headache remains to be fully established.

For these reasons, the goal of this work is to investigate the possible role of ophthalmologists in headache evaluation in a third-level pediatric teaching hospital and determine which diagnostic investigations are recommended.

## 2 Materials and methods

### 2.1 Study design

This is a prospective monocentric study conducted at the Institute for Maternal and Child Health—IRCCS “Burlo Garofolo” of Trieste, Italy, from 1st October 2023 to 30th April 2024.

As assessed in other studies, 100 patients were considered a representative sample as they had already been selected as a secondary-level group (12).

### 2.2 Inclusion criteria

All children aged 4–18 who presented to a secondary-level paediatric outpatient clinic with unresolved headaches (defined as headache non-responding to symptomatic treatment) or headaches exhibiting urgent characteristics (defined as worsening pattern, lack of response to treatment, vomiting, neurological symptoms) were referred to the ophthalmology department.

### 2.3 Exclusion criteria

Patients with traumatic headaches, infectious disease, intellectual disability and very recent onset (presence of symptoms for less than 2 weeks) without red flags for a possible life threatening headache (12, 13) were excluded.

### 2.4 Methods investigation

**Figure 1:**
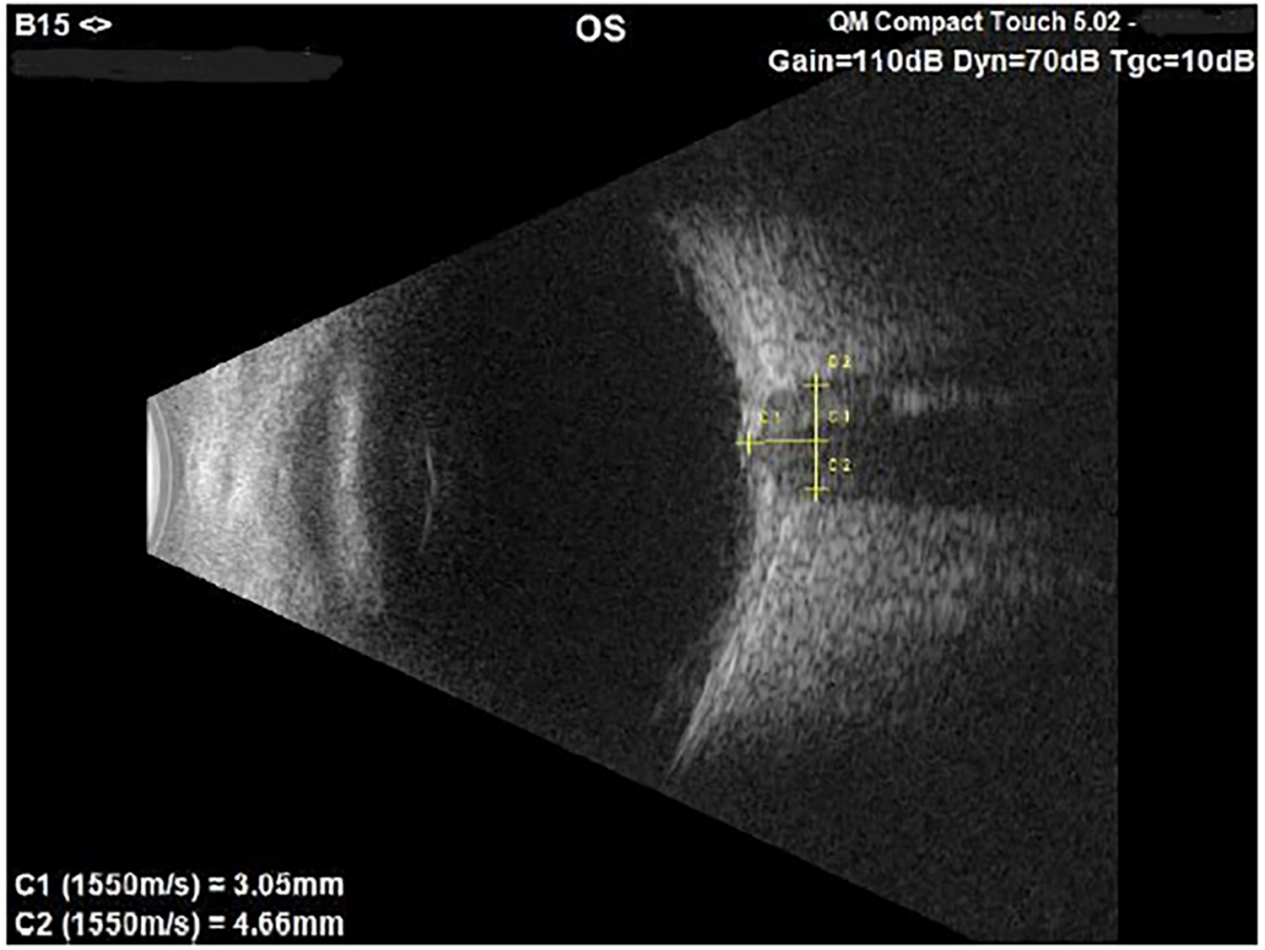
B-scan image of the left optic nerve in a 7-year-old male suspected of papilledema. The ONSD measures 4.66 mm, value above the norm compared to our standard.

All patients underwent the following investigations:

- A questionnaire regarding the localization and duration of the headache, the time of day when it appeared, any previous or ongoing pathologies, including ocular ones, and the use of electronic devices (Supplementary Table S1).
- Complete ophthalmologic examination with cycloplegic refraction;
- Orthoptic evaluation;
- Colour and autofluorescence retinography [Topcon TRC 50 DX] to evaluate the optic disc;
- Optical coherence tomography (OCT) [Optovue RTVue XR 100] to evaluate the subfoveal choroidal thickness, the retinal nerve fibres layers (RNFL) and optic nerve head (ONH);
- Ocular ultrasound B-scan [Quantel Medical Compact Touch, 15 MHz probe] to assess the optic disc and the Optic Nerve Sheath Diameter (ONSD). The Compact Touch is a portable instrumentation which can therefore also be used in emergency room. The probe was placed on closed eyelids, using an ultrasonography gel, and when the optic nerve was aligned opposite to the probe, the image of the optic nerve was saved. The off-line measurement of the ONSD was then performed 3 mm behind the optic disc (Figure 1).

To classify the cases of headache we adhered to the International Classification of Headache Disorders (7). Regarding the ophthalmic forms, we also considered the headaches related to strabismus and those to digital eye strain (DES) only if all other forms were ruled out. If necessary, the diagnosis was confirmed using instrumental investigations, including magnetic resonance imaging and lumbar puncture. Magnetic resonance imaging (MRI) was performed in all patients with ONSD >4.5 mm in at least one eye, who underwent also a neurological examination. Neurological evaluation was performed as well in patients without an ophthalmic-related headache or another diagnosis of secondary headache.

In HARE and DES, the ophthalmological diagnosis was confirmed by the disappearance of the headache after correction of the refractive error (found for the first time or no longer adequate) or by reducing the application on electronic instruments to 1 h a day. The reassessment was conducted after two months.

Regarding refractive defects (14), a myopic patient was defined as a patient with a spherical equivalent refraction (SE) of at least −0.50 diopters (D) even if in just one eye; a hyperopic patient if hyperopia (SE) of +2 D or more was present at least in one eye; an astigmatic patient if a cylinder more than 1 D was present at least in one eye; an anisometropic patient when the SE difference between the two eyes was at least 2 D.

Regarding strabismus, we performed the cover test with prisms, for the estimation of the size of deviation, for near and for distance, both unilateral cover test (or cover-uncover test) and alternating cover test to detect the type of deviation, manifest or latent; and stereopsis test (Titmus stereo test) to confirm the perfect ocular alignment at least for near.

To alleviate the symptoms, we conducted orthoptic exercises in cases of convergence deficit; in cases of intermittent and manifest exotropia, performing surgical interventions when necessary; in cases of accommodative spasm, we used atropine 1% eye drops for months [in these more serious cases drops are given once a day, in the morning, and are suspended after 2 months. If the spasm persists, it is performed for another 2 months (15)] and during this period bifocal glasses are prescribed with cycloplegic refraction until the spasm was completely resolved.

The study was approved by the IRCCS Burlo Garofolo Institutional Review Board (IRB) using the 34–18 protocol, all parents signed a written consent for anonymous use of data.

### 2.5 Statistical analysis

Data were collected in an anonymized electronic database specifically developed for the study. Categorical variables were described through absolute and percentage frequencies, continuous variables were described by means and standard deviations or medians and interquartile ranges, according to their distribution.

Univariate and multivariate linear models were fit to investigate associations between variable of interest (age vs. Choroidal Thickness, Refractive Error vs. Choroidal Thickness and RNFL Average). The Kruskal–Wallis test and the Wilcoxon-Mann–Whitney test were used to compare two samples. All the tests were two-tailed and a *p*-value of <0.05 was considered as statistically significant. The statistical software Stata 18 was used for statistical analysis.

## 3 Results

One hundred-one patients with headaches were enrolled in the study. No patient refused or did not participate in follow-up. Females were 59 (58.4%) and males 42 (41.6%). The population's average age was 11.3 years, with a standard deviation (SD) of 3.3 years (range: 4–18 years). All children underwent the ophthalmological evaluation. Fifty-four (53.5%) received a diagnosis of secondary headache, while the other 47 (46.5%), at the end of the evaluation performed by a child neurologist, had a diagnosis of migraine or TTH.

**Figure 2:**
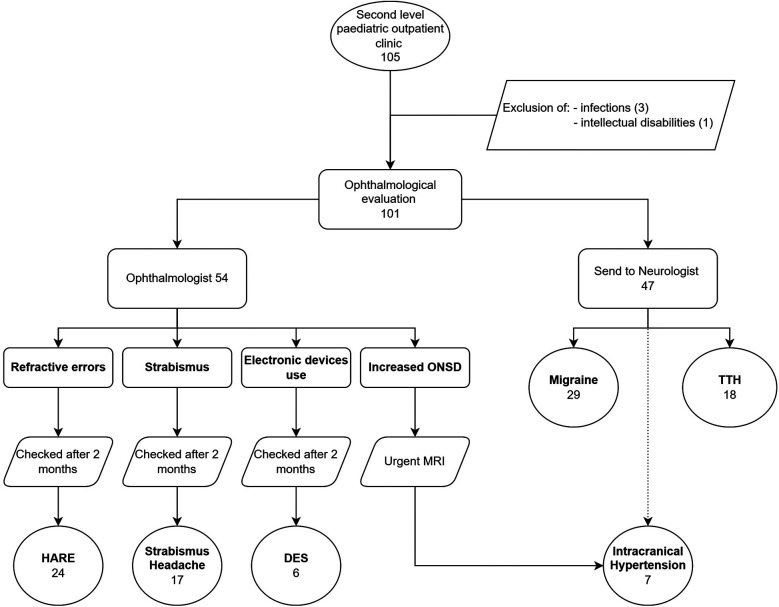
Descriptive chart of the process followed in our study sample.

Patients were consequentially distributed into 6 categories (Figure 2).

Regarding the questionnaire, types and localizations of headaches are described in Table 1.

**Table 1:** Description of types and localizations of headaches divided into the 6 categories.

General characteristics	Characteristics	Migraine		TTH		IH		HARE		Strabismus		DES	
		*N*	%	*N*	%	*N*	%	*N*	%	*N*	%	*N*	%
Type	Throbbing	17	58.62	4	22.2	1	14.28	9	39.13	5	27.7	3	50
	Compressive	8	34.78	10	55.5	3	42.86	10	43.47	9	50	3	50
	Imprecise	4	13.79	4	22.2	4	57.14	4	17.4	4	22.2	0	0
Localization	Frontal	7	24.14	9	50	2	28.57	10	43.47	7	38.8	2	33.3
	Bitemporal	8	27.58	5	27.7	0	0	6	26	0	0	2	33.3
	Holocranial	6	20.69	3	16.6	4	57.14	5	21.74	3	16.6	1	16.6
	Monolateral	4	13.79	0	0	0	0	0	0	0	0	0	0
	Occipital	0	0	0	0	1	14.28	0	0	3	16.6	1	16.6
	Unspecific	4	13.79	1	5.5	0	0	2	8.7	5	27.7	0	0

### 3.1 Migraine

In the 29 migraine patients three OCT parameters were studied, following scientific literature results:

#### 3.1.1 Choroidal thickness

To evaluate whether variations of OCT choroidal thickness are present in children migraine during attack-free period, we first assessed its age dependence.

**Figure 3:**
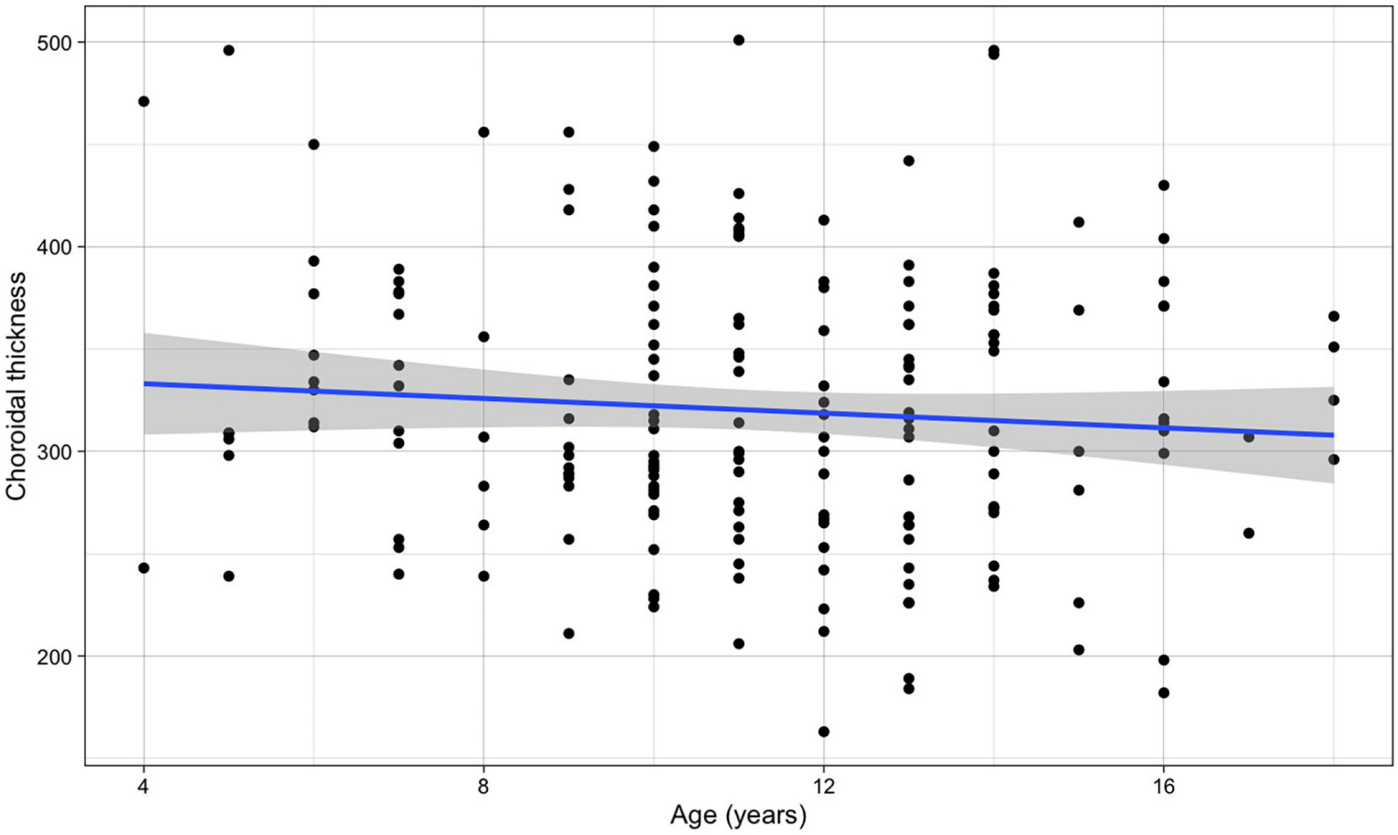
Relationship between choroidal thickness and age (the blue line refers to the regression line for a simple linear regression, *β* = −1.796, *p*-value = 0.265).

No significant association was observed between choroidal thickness and age across the pediatric population studied (β for simple linear regression = −1.796, *p*-value = 0.265) (Figure 3).

No difference was found between the right eye and the left eye (*p*-value = 0.594). Therefore, we carried out further evaluations with all eyes together.

**Figure 4:**
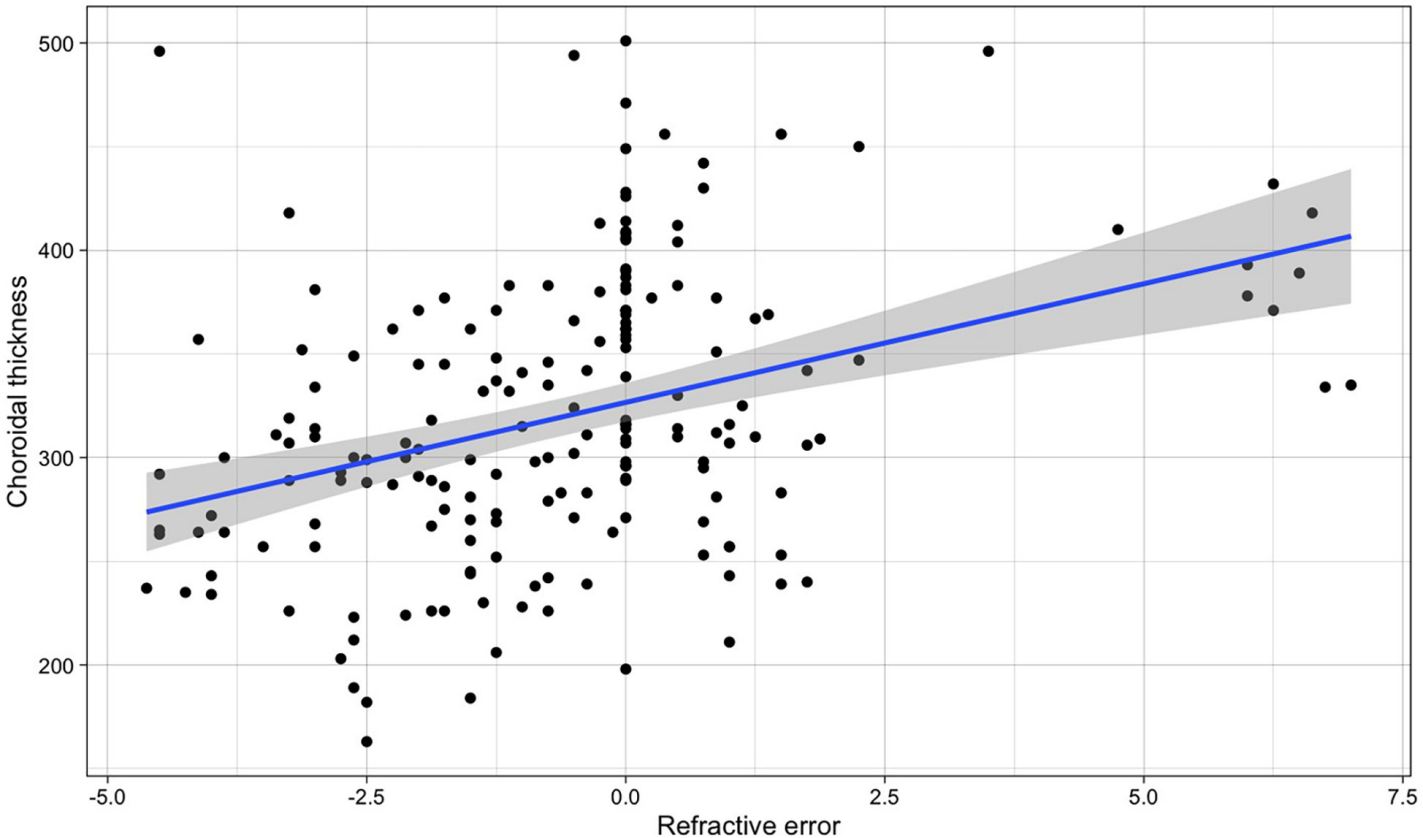
Relationship between refractive error and choroidal thickness in both eyes in the whole population (the blue line refers to the regression line for the simple linear regression, *β* = 11.44, *p*-value < 0.001).

Instead, a clear variation with refraction (with lower values in myopic patients and higher in hyperopic) (Figure 4) was found (β for simple linear regression = 11.44, *p*-value < 0.001). When correcting for age in a multiple linear regression, the association with refraction was very similar (*β*_SE_ = 11.86, *p* < 0.001), while age resulted as not associated (*β*_age_ = 0.925, *p* = 0.560). Therefore, we compared the values of migraine patients with those of the other children with the same refraction range (between 0 and +1.50 D).

The mean value of choroidal thickness in migraine patients was 320 (±56) µm and showed a statistically significant difference (*p*-value = 0.001) with the thickness of the children without migraine that was 362 (±66) µm.

#### 3.1.2 RNFL

**Figure 5:**
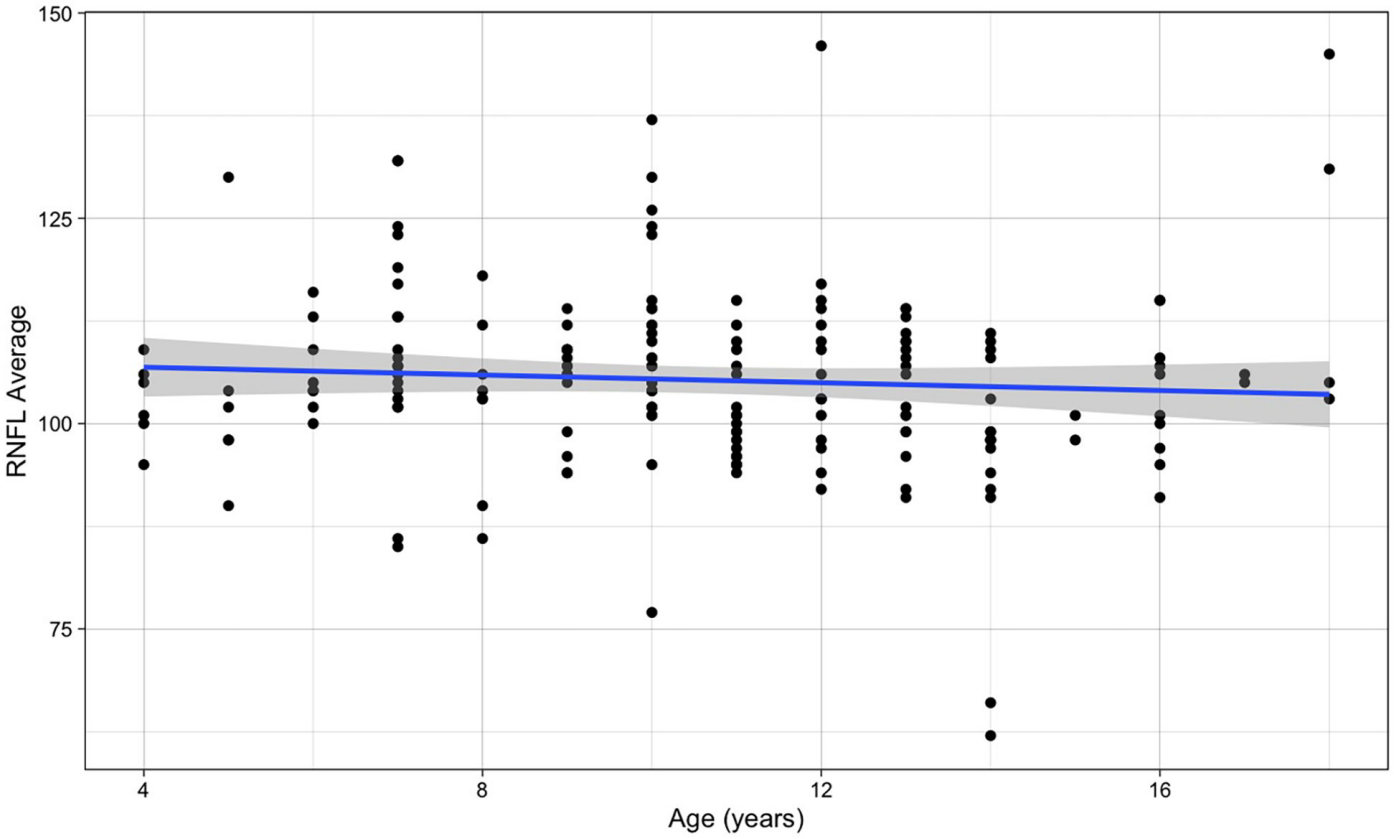
Relationship between RNFL average and age (the blue line refers to the regression line for a simple linear regression, *β* = −0.236, *p*-value = 0.344).

It was found no significant association between thickness of the RNFL and age (β for simple linear regression = −0.236, *p*-value = 0.344) (Figure 5). Also RNFL values for the right and left eye were similar (*p*-value = 0.653).

The mean value of both eyes in migraine was 102.5 (±6.8) µm, vs. the 106.1 (±11.6) µm of the other children; a small reduction, but not statistically significant (*p*-value = 0.078). This result remained similar when considering the upper and lower sectors separately. The superior sector showed values of 104.7 (±7.3) µm in migraine and 107.4 (±11.4) in controls (*p*-value = 0.136), the inferior sector 101.3 (±7.3) µm against 104.3 (±15.6) µm (*p*-value = 0.205) (Table 2).

**Table 2:** RNFL thickness patients with migraine compared with the others.

	Migraine		
Variables, average (±S.D.) µm	No *N* = 72	Yes *N* = 29	*p*-value
RNFL thickness average	106.1 (±11.6)	102.5 (±6.8)	0.078
RNFL thickness superior	107.4 (±11.4)	104.7 (±7.3)	0.136
RNFL thickness inferior	104.3 (±15.6)	101.3 (±7.3)	0.205

#### 3.1.3 ONH

As regards the Optic Nerve Head (ONH), a significant difference was found in the cup/disc parameters of children with and without migraine, both in the right and left eye without difference.

The three considered parameters were the cup/disc (c/d) Area ratio, the c/d Volume (V) ratio and the c/d Height (H) ratio.

Considering both eyes together, the average c/d Area ratio in migraine was 0.18 (±0.1), compared to 0.13 (±0.14) of the controls (*p*-value = 0.011), the average c/d V ratio was 0.34 (±0.18) in migraine and 0.23 (±0.22) in the others (*p*-value = 0.002), and the average c/d H ratio was 0.43 (±0.21) for migraine, vs. 0.30 (±0.27) of the other children (*p*-value = 0.002) (Table 3).

**Table 3:** Cup/disc analysis in patients with migraine compared with the others.

	Migraine		
Variables, average (+S.D.)	No *N* = 72	Yes *N* = 29	*p*-value
ONH analysis c/d, Area ratio	0.13 (±0.14)	0.18 (±0.1)	***0.011***
ONH analysis c/d, Volume ratio	0.23 (±0.22)	0.34 (±0.18)	***0.002***
ONH analysis c/d, Height ratio	0.30 (±0.27)	0.43 (±0.21)	***0.002***

*p*-value in bold and italics for all statistically significant cases (<0.05).

### 3.2 Tension type headache

In the 18 patients with a final diagnosis of TTH, no OCT or ultrasound parameters evaluated in the study showed any statistically significance difference with the other patients.

### 3.3 Intracranial hypertension

Of the 7 patients with Intracranial Hypertension, 5 had idiopathic intracranial hypertension, one a Chiari 1 malformation and one a brain tumor (pilocytic astrocytoma).

In all 101 patients with headache, the fundus oculi and the ultrasound, especially for ONSD, were evaluated. A bilateral evident papilledema resulted in 5 patients (71.43%), whereas the other 2 patient of this group did not manifest it. But in all 7 patients resulted an increased value of the ONSD. About the patients without papilledema one was the patient with the pilocytic astrocytoma and he had an ONSD of 5.05 mm in the right eye and 5.95 mm in the left eye. The second patient without papilledema was the one with the Chiari 1 malformation, the ONSD of the right eye was 4.52 mm and 4.65 mm in the left eye.

Patients with intracranial hypertension presented a ONSD median of 5.2 (RE, 5.1–6.2) mm and 5.5 (LE, 5.1–6.0) mm, in the others was 4.5 (RE, 4.1–4.7) mm and 4.4 (LE, 4.2–4.8) mm. The difference was significant for both right (*p* = 0.003) and left (*p* = 0.007) eyes.

This difference was statistically significant also for the mean values of both eyes (*p*-value < 0.001): the average was 5.6 (±0.59) mm in Intracranial Hypertension and 4.5 (±0.54) mm in the rest of the population (Table 4).

**Table 4:** ONSD parameters compared in patients with and without Intracranial Hypertension.

	Intracranial hypertension		
Variables	No *N* = 94	Yes *N* = 7	*p*-value
ONSD RE, median (IQR)	4.5 (4.1–4.7) mm	5.2 (5.1–6.2) mm	***0.003***
ONSD LE, median (IQR)	4.4 (4.2–4.8) mm	5.5 (5.1–6.0) mm	***0.007***
ONSD, average (±SD)	4.5 (±0.54) mm	5.6 (±0.59) mm	***<0.001***

*p*-value in bold and italics for all statistically significant cases (<0.05).

### 3.4 HARE

HARE was found in 24 children. The refractive error (SE) was myopia (range −0.50/−2.25 D) in 12 patients and hyperopia (range +2.00/+6.50 D) in 6. Nine patients had astigmatism (range 1.00/3.50 D), three of which were also assigned to the hyperopic category. No anisometropic patient has been found.

One patient experienced resolution of his symptoms with the application of glasses with very low cylindrical lenses, less than 1.00 D: he had a hyperopic compost astigmatism of +0.50 sph and +0.50 cyl/85 in both eyes.

### 3.5 Strabismus

Strabismus was found in 17 children. One displayed a manifest form (exotropia of −16 prism dioptres, PD, for near and distance), 7 (41.2%) had intermittent exotropia, 6 (35.3%) had convergence insufficiency, 2 had accommodative spasm and 1 had a non-accommodative convergence excess.

### 3.6 DES

Headaches attributed to digital eye strain (DES) were identified in 6 patients, and in 4, they were accompanied by photophobia.

The commonly used electronic devices were the same proportion (33%) as television, video games, and mobile phones. Videogames were also used in the evening before bedtime.

The range of daily time spent in front of electronic devises was 2 to 9 h (average of 5 ± 2.7). Reducing their use to one hour per day resolved the headache.

## 4 Discussion

This study demonstrated that an ophthalmic evaluation allowed significant diagnoses in children with headaches without a history of trauma and who did not present inflammatory states of the respiratory tract or the otolaryngological and dental fields.

The ophthalmic causes of secondary headaches represented as much as 46.5% of cases, the most frequent among them was HARE, with a frequency of 23.7%. Prevalence studies led to conflicting results. Some reported a frequency of 26.1% of ocular causes for headaches, with 18.2% caused by refractive issues (8). Studies performed in ophthalmology departments found higher prevalence, with almost two-thirds of children presenting with HARE (16, 17). Others, among hospitalized patients for headaches, reported that only 1.7% of cases were caused by HARE (18). Our data, evaluated on the population derived from both emergency room and outpatient settings, produce values that are halfway between those just reported.

*Akinci* (14) identified a higher prevalence of refractive errors in the population with headaches compared to that without (14). In schoolchildren with headache *Hendricks* (19) detected more spherical refractive errors in females and cylindrical errors in males. In our sample, this difference was not evident.

The beta version of the International Classification of Headache Disorders (ICHD) (20) included headaches caused by strabismus, but it was missing from the final versions. Studies of prevalence demonstrated that headaches due to new or worsening strabismus had a frequency of 4.4% and 25.3% of those with new strabismus had convergence insufficiency (8). In our study the 16.3% of children had headache caused by strabismus and of these one third had convergence insufficiency; our data show a higher frequency of strabismus. Consequently, we believe that strabismus should be included among the secondary causes of headache in children.

Intracranial hypertension was identified in 6.9% of patients, a higher prevalence than that reported in other studies, which typically found rates around 3% (8). This difference was probably due to the selection of population examined.

Finally, the 5.9% of headaches experienced were associated with digital eye strain (DES). This percentage had increased compared to the pre-COVID era, although it was lower than during the lockdown period when it was around 50% (11).

Given that spontaneous transient retinal vasospasm has been reported in some patients as a possible cause of retinal migraine, that the reduction of flow in the posterior ciliary arteries could be associated with dysregulation of choroidal flow (7, 21, 22), we evaluated the OCT choroidal thickness in all children. Age and refractive error are known to influenced choroidal (21, 22). However, no age variation was detected in our group, while significant variations were observed with refraction, also for minor refractive variations (Figure 4).

In adult migraine subjects, while a reduction in choroidal thickness (23–25) was noticed during attack-free period, an increase in it was observed during acute migraine attacks (26). In the paediatric population there are few studies available. *Nalcacioglu* (27) did not reveal significant differences, unlike our case, which considered all the children. The choroidal thickness of our children, for migraineurs measured during attack-free periods, was significantly lower in migraine (*p*-value 0.001) only if the child's refractive error was evaluated. Being children, it was also likely that with the repetition of vasospasm episodes in migraine, this difference became even more important.

We must emphasize the importance of knowing the refractive status of patients and controls in all studies of choroidal thickness.

Age influenced the variations in RNFL thickness values (28), but very few data have been acquired in the paediatric population.

*Lin* (29), in his meta-analysis of 26 studies, discovered that in adults the RNFL was thinner in patients with migraines, especially those with aura. In contrast, in the only study dedicated to the paediatric population, *Nalcacioglu* (27) registered a non-significant difference. Our results also demonstrated a reduction of RNFL (102.5 vs. 106.1 µm), but not noteworthy (*p* = 0.078). It could be that multiple migraine attacks over the years have made the difference substantial.

Some Authors highlighted an increased risk for normal-tension glaucoma in the adult migraine population and a potential common vascular aetiology of both diseases (6, 7, 30, 31). Since low-pressure glaucoma is diagnosed through the reduction of RNFL, together with the increase in papillary excavation and of the cup/disc ratio (C/D) of the ONH, we also evaluated the C/D. We considered it pathological when the C/D Area was higher than 0.20–0.30 (29, 32). In our patients it remained below 0.3, with an average of 0.18 in migraine vs. 0.13, but with a significant difference (*p*-value 0.011).

Given the presence of both a slight decrease in RNFL and a significant elevation in the C/D in our paediatric migraine population, we advise a periodic assessment of Optical Coherence Tomography (OCT) parameters in these patients (5). We believe that further studies in the migraine population will be necessary to clarify their significance.

Intracranial hypertension has always been a topic of great interest for ophthalmologists. In children, the intensity of headaches was not correlated with the severity of the condition (33), and the sole assessment of the ocular fundus was not sufficiently sensitive in identifying it. Indeed, the paediatric population exhibited a latency in the manifestation of papilledema, for this reason ultrasonographic evaluation of ONSD must be considered a fundamental examination (34, 35). In our population, all 7 patients with intracranial hypertension had ONSD increased (*p* < 0.001), while only 5 (71.4%) exhibited papilledema at the time of their ophthalmic evaluation. Given the non-invasive nature and high sensitivity of this examination, we emphasized the importance of its use in patients suspected of intracranial hypertension without a visible papilledema. Some authors propose the use of ocular ultrasound in the emergency room at the patient's bedside using the POCUS (with 7.5 MHz probe) without the help of an ophthalmologist. In this perspective, some concerns should be taken into account, both about the personnel employed and the probe that allows a lower resolution than the 15 MHz one we use (36). Other authors have proposed the use of standardized A-scan ultrasound, which employs an 8 MHz probe, for the evaluation of ONSD (37–39). While the standardized A-scan technique offers more precise ONSD measurement, it requires significant training and involves direct contact with the eye after anesthetic administration, with the patient maintaining a fixed gaze—making it unsuitable for children or uncooperative patients. Our study aims not to use the most precise method, but rather one that is better tolerated by children and allows assessment of both ONSD and optic disc drusen with minimal cooperation.

Overall, forty-seven patients (46.5%) presented with headache which could be surely related to ophthalmic conditions. According to *Thorud* (40), a headache due to HARE is generally frontal (43.47% of our cases; Table 5) and more likely during school hours (70.8%).

**Table 5:** Causes of headache in the whole population.

Population	Migraine		TTH		Intracranial hypertension		HARE		Strabismus		DES	
	*N*	%	*N*	%	*N*	%	*N*	%	*N*	%	*N*	%
F (59)	15	25.4	9	15.2	4	6.7	14	23.7	12	20.3	4	6.7
M (42)	14	33.3	9	21.4	3	7.1	10	23.8	5	11.9	2	4.7
TOT (101)	29	28.7	18	17.8	7	6.9	24	23.7	17	16.8	6	5.9

F, female; M, male; TTH, tension-type headache; HARE, headache attributed to refractive errors; DES, digital eye strain.

Regarding the pathogenesis of headache, *Lajmi* (41) reported that in hyperopic patients the pain was a consequence of the prolonged accommodative effort and in myopia it results from scalp and periorbital muscle contraction with narrowing of the palpebral fissures, both leading to headaches similar to tension-type (41). *Mohan* (11) instead found that the most common location of headache was frontal among children with myopia but never in children with hyperopia or astigmatism, where occipital headache was more frequent.

In our cases, myopia was the most prevalent type of HARE, accounting for 50% of cases, often of mild severity (below 3 D), more frequent than astigmatisms and hyperopia, traditionally considered the main causes of HARE (40). No child reported occipital pain.

It is therefore necessary to correct even slight refractive defects to prevent headaches, also because they can act as triggers for migraine (42).

Regarding strabismus, it is more often associated with frontal pain (10), as in our cases (38.8%; Table 5). Blurred vision, diplopia, loss of position during reading, tearing, eye or orbit pain, and loss of concentration were common symptoms (10). According to *Lin* (8), who reported that the 25.3% of the newly diagnosed strabismus-related headaches had convergence insufficiency, our findings are of similar (35.3%). Convergence insufficiency could also act as a trigger for migraine attacks, appearing shortly after near application (9).

Among our children, most of cases (40%) presented intermittent exotropia without convergence insufficiency which needed surgical or rehabilitative interventions to resolve headaches. This type of strabismus did not cause significant disturbances (43), but our study did not confirm these results.

Regarding DES, no particular electronic device currently appeared more likely to cause headaches than another (44). We identified DES only in six patients, and this small number did not allow us to understand which device was predominantly associated. Most of the literature suggested that excessive duration was when daily usage exceeds as little as two hours in children over the age of 2 years (16, 41–44) and in our children it was 2–9 h.

The use of devices before bedtime has also been associated with a higher risk of developing headaches (45), and this finding also emerged in our study, where 4 subjects used mobile video games before going to sleep. This headache is probably linked to sleep disorders resulting from this bad habit: in fact, for children aged 3–6 years the association between TV viewing time and the risk of sleep disorder is known (46). Spending more time on viewing screens reduces the time spent on other activities, such as sleep, and especially exposes children to blue light, which delays sleep onset and reduces sleep quality (47). Shorter sleep duration due to watching TV or using computer, produces children being tired in school, and having difficulties both in waking up and in sleeping (48).

*Lajmi* (41) highlighted that prolonged exposure to electronic devices could provoke headaches even if the refractive errors were corrected, as in our 6 cases. It is under study whether electronic device distance is associated with the development of myopia (45, 48).

The locations and types of pain present in patients with primary headaches were well reported; with this work we added some data on the type of pain present in headaches secondary to ocular pathology. As in tension-type headache, the frontal localization and the compressive type appeared to be the most frequent in ophthalmic conditions (Table 1).

This study has some limitations. The most important is the selection of cases through a second-level headache clinic, which resulted in a smaller patient sample; on the other hand, this simplified a standardized evaluation procedure. The strengths of our study include the comprehensive ophthalmologic assessments conducted on all patients and the diagnostic confirmation provided by the paediatric neurologist.

## 5 Conclusion

This study shows that many children with headaches have purely ophthalmic problems. Furthermore, it reminds physicians that all ophthalmological causes of headaches can trigger the onset of migraine attacks.

Finally, these data suggest that an optic nerve ultrasound should accompany the fundus observation for a correct and early indication of intracranial hypertension.

## Data Availability

The raw data supporting the conclusions of this article will be made available by the authors, without undue reservation.
