# Supplementary material for: Headache in the paediatric population: the role of the ophthalmologist
Source: Front Pediatr. 2025 Jun 12;13:1547750. doi: 10.3389/fped.2025.1547750 (PMC12198240; doi:10.3389/fped.2025.1547750)
Supplement: Supplementary file 1 [file Datasheet1.pdf]

	QUESTIONS	Answers
1	What is the time pattern of your headache	A. Sudden first headache<br>B. Episodes of headache<br>C. Daily headache<br>D. Gradually worsening headache<br>E. Mixture
2	How and when did your headache(s) begin?	
3	How often do your headaches occur?	
4	Do you have more than 1 type of headache?	
5	Are there warning signs or can you tell that a headache is coming?	
6	How long do they last?	
7	Where is the pain located?	Location:<br>A. Frontal<br>B. Bitemporal<br>C. Holocranial<br>D. Monolateral<br>E. Ocipital<br>F. Unspecific
8	How would you describe your headache?	Character:<br>A. Throbbing<br>B. Compressive<br>C. Stabbing<br>D. Other/Imprecise
9	Are there any other symptoms that accompany your headache? (Nausea, vomiting, dizziness, numbness, weakness)	
10	Have you ever experienced visual obscuration during headache?	A. Yes<br>B. No
11	Have you ever experienced double vision during a headache?	A. Yes<br>B. No
12	Have you ever experienced photophobia during a headache?	A. Yes<br>B. No
13	Have you ever experienced phonophobia during a headache?	A. Yes<br>B. No
14	Does the headache increase on bending /coughing?	A. Yes<br>B. No
15	Is there any redness of eyes/watering/running nose/drooping of eyelid during the headache?	A. Yes<br>B. No
16	What makes the headache better?	
17	What makes the headache worse?	
18	What do you do when you get a headache? Do you have to stop your activities when you get a headache?	
19	How much time do you spend on screen time on a daily average? (including mobile, television, gaming devices and computers)	

20	Which device do you use the most?	A. Mobile phone<br>B. Television<br>C. Gaming devices<br>D. Computer<br>E. Tablet
21	Do you play video games?<br>If so, do you sometimes play them before going to sleep?	
22	Have you noticed a worsening of your headache after using electronic devices?	
23	Do the headaches occur under any special circumstances or at any particular time?	
24	Do you have any other medical problems?	
25	Does anyone in your family suffer from headaches? Do you know which kind od headache it is?	
